# A Novel Role for Adipose Ephrin-B1 in Inflammatory Response

**DOI:** 10.1371/journal.pone.0076199

**Published:** 2013-10-01

**Authors:** Takuya Mori, Norikazu Maeda, Kana Inoue, Ryohei Sekimoto, Yu Tsushima, Keisuke Matsuda, Masaya Yamaoka, Takayoshi Suganami, Hitoshi Nishizawa, Yoshihiro Ogawa, Tohru Funahashi, Iichiro Shimomura

**Affiliations:** 1 Department of Metabolic Medicine, Graduate School of Medicine, Osaka University, Suita, Osaka, Japan; 2 Departments of Organ Network and Metabolism, Graduate School of Medical and Dental Sciences, Tokyo Medical and Dental University, Tokyo, Japan; 3 Departments of Molecular Endocrinology and Metabolism, Graduate School of Medical and Dental Sciences, Tokyo Medical and Dental University, Tokyo, Japan; 4 Departments of Metabolism and Atherosclerosis, Graduate School of Medicine, Osaka University, Suita, Osaka, Japan; University of Sao Paulo, Brazil

## Abstract

**Aims:**

Ephrin-B1 (*EfnB1*) was selected among genes of unknown function in adipocytes or adipose tissue and subjected to thorough analysis to understand its role in the development of obesity.

**Methods and Results:**

*EfnB1* mRNA and protein levels were significantly decreased in adipose tissues of obese mice and such reduction was mainly observed in mature adipocytes. Exposure of 3T3-L1 adipocytes to tumor necrosis factor-α (TNF-α) and their culture with RAW264.7 cells reduced EFNB1 levels. Knockdown of adipose EFNB1 increased monocyte chemoattractant protein-1 (*Mcp-1*) mRNA level and augmented the TNF-α-mediated THP-1 monocyte adhesion to adipocytes. Adenovirus-mediated adipose EFNB1-overexpression significantly reduced the increase in *Mcp-1* mRNA level induced by coculture of 3T3-L1 adipocytes with RAW264.7 cells. Monocyte adherent assay showed that adipose EfnB1-overexpression significantly decreased the increase of monocyte adhesion by coculture with RAW264.7 cells. TNF-α-induced activation of extracellular signal-regulated kinase 1/2 (ERK1/2) was reduced by EFNB1-overexpression.

**Conclusions:**

EFNB1 contributes to the suppression of adipose inflammatory response. In obesity, reduction of adipose EFNB1 may accelerate the vicious cycle involved in adipose tissue inflammation.

## Introduction

Obesity, especially visceral fat obesity, is an important aspect of the metabolic syndrome and atherosclerosis [1,2]. In the Human Body Map project [3], our group provided evidence for the endocrine function of adipose tissue, in addition to serving as an energy storage organ [4]. Our group also discovered *Adiponectin* among human adipose tissue cDNAs [5]. Clinical evidence indicates that adipocytes produce various cytokines and chemokines, which we named adipocytokines, and that the obesity-related changes in adipocytokines contribute to the development of the metabolic syndrome [6]. Infiltration of immunocytes, such as macrophages, is observed in obese adipose tissues and these cells induce chronic low-grade inflammation by producing a battery of inflammatory cytokines and chemokines [7-9]. Moreover, the infiltrated macrophages interact with adipocytes via inflammatory mediators, such as free fatty acids and adipocytokines, to generate a metabolic vicious cycle, which eventually lead to the clinical spectrum of the metabolic syndrome [10].

It is our view that the visceral fat status affects and reflects the gene expression profile in peripheral blood cells. In this regard, we reported recently the association between visceral fat adiposity and gene expression profile of peripheral blood cells in human subjects [11,12]. Furthermore, in a series of exploratory research studies, we searched for genes of unknown function in adipocytes and adipose tissues by comparing the cDNA microarray-based gene expression patterns of human peripheral blood cells and mouse adipose tissues. Our search identified various genes, among them ephrin-B1 (*EfnB1*). Historically, the first Eph receptor, EphA1 and its Eph receptor-interacting (ephrin) ligand, ephrin-A1, were cloned from cancer cells [13,14]. Increasing evidence indicate that Eph receptors and their ephrin ligands mainly serve as a cell communication system and their interactions play crucial roles in both normal steady conditions and pathological diseases [15]. A unique feature of the Eph-ephrin complexes is their capability to transduce bidirectional intracellular signals; such property closely regulates the physiological and pathological cellular events as well as developmental processes [16].

To our knowledge, there is virtually no information on the regulation and functions of EFNB1 in adipocytes. In this report, we describe the novel role of EFNB1 in the development of adipose inflammation.

## Materials and Methods

### Animals

Male C57BL/6N mice and *ob/ob* mice were obtained from Charles River Japan Inc. (Kanagawa, Japan) and maintained at 22°C under a 12:12-h light–dark cycle (lights on from 7:00 to 19:00). For analysis of tissue distribution, 12-week-old male C57BL/6N mice were euthanized by bleeding from the inferior vena cava under anesthesia after 12 hrs of fasting, and various tissue samples were excised. For the diet-induced obese (DIO) model study, 8-week-old male C57BL/6N mice were fed either regular chow diet (MF; Oriental Yeast, Osaka, Japan) or high-fat and high-sucrose (HF/HS) diet (F2HFHSD; Oriental Yeast) for 8 weeks. At 16 weeks of age, the mice were euthanized under feeding condition, blood samples were collected from the inferior vena cava, and epididymal white adipose tissues (WAT) were excised. For obese model mice study, C57BL/6N and *ob/ob* mice were fed regular chow diet and sacrificed at 8 or 16 weeks of age. In all experiments, mice were anesthetized with an intraperitoneal injection of a mixture of medetomidine (0.3 mg/kg body weight), midazolam (4 mg/kg body weight) and butorphanol tartrate (5 mg/kg body weight). The experimental protocols were approved by the Ethics Review Committee for Animal Experimentation of Osaka University School of Medicine. This study also conforms to the Guide for the Care and Use of Laboratory Animals published by the US National Institutes of Health.

### Fractionation of WAT

WAT were minced in Krebs-Ringer buffer [composition: 120 mmol/L NaCl, 4 mmol/L KH_2_PO_4_, 1 mmol/L MgSO_4_, 1 mmol/L CaCl_2_, 10 mmol/L NaHCO_3_, 30 mmol/L HEPES, 20 mmol/L adenosine, and 4% (wt/vol) bovine serum albumin (Calbiochem, San Diego, CA)]. Tissue suspensions were centrifuged at 500 x *g* for 5 min to remove erythrocytes and free leukocytes. Collagenase was added to a final concentration of 2 mg/mL and suspensions were incubated at 37°C for 20 min under continuous shaking. The cell suspension was filtered through a 250 µm filter and then spun at 300 x *g* for 1 min to separate the floating mature adipocytes fraction (MAF) from the stromal vascular cell fraction (SVF) pellet. This fractionation and washing procedures were repeated twice with Krebs-Ringer buffer. Finally, both fractions were washed with phosphate buffered saline (PBS) and subjected to quantitative real-time polymerase chain reaction (RT-PCR).

### Cell cultures

3 T3-L1 adipocytes and RAW264.7 macrophages were maintained as described previously [17]. 3T3-L1 adipocytes were treated with the indicated concentrations of tumor necrosis factor-α (TNF-α) for 24 hrs, harvested, and then subjected to quantitative RT-PCR. Cocultures of 3T3-L1 adipocytes and RAW264.7 cells were prepared using a separate system as described previously [10]. Briefly, on day 8 after the induction of differentiation, 3T3-L1 adipocytes in 6-well plates were co-cultured with RAW264.7 cells (1.0×10^5^ or the indicated number of cells) using transwell inserts with a 1 µm porous membrane (BD Falcon, MD) (Figure S1). After incubation for 24 or 48 hrs, 3T3-L1 adipocytes and RAW264.7 cells were harvested and subjected to quantitative RT-PCR or immunoblotting.

### Introduction of siRNA

On day 7 after the induction of differentiation, 3T3-L1 adipocytes were transfected with siRNA for *EfnB1* (forward sequence 5’-CUA UGA AGA UGU UAU GAA TT-3’ and reverse sequence 5’-UUC AUA ACG AUC UUC AUA GTG-3’, Qiagen, Valencia, CA) using DeliverX Plus siRNA Transfection kit (Affymetrix, Santa Clara, CA) according to the protocol recommended by the manufacturer. The transfected cells were incubated for 24 hrs and then treated with the indicated concentrations of TNF-α. After treatment with TNF-α for 24 hrs, the cells were harvested and subjected to quantitative RT-PCR or western blotting. In these experiments, allstars negative control siRNA (Qiagen) was used as a control.

### Construction and preparation of Ephrin-B1-expressing adenovirus

The full-length cDNA of *EfnB1* from mouse colon was subjected to RT-PCR using *Pfu* DNA polymerase (Promega, Madison, WI) with primers containing a restriction enzyme cutting site at the end. The amplicons were cloned into pENTRTM1A vector (Life Technologies, Carlsbad, CA) using restriction enzyme sites. After confirming the correct sequences, the genes encoding ephrin-B1 in pENTRTM1A vector were transferred into the adenoviral expression vector (pAd/CMV/V5-DEST; Life Technologies) by recombination following the instructions provided by the manufacturer. The resultant pAd/CMV plasmids containing the target gene were linearized by *Pac*I digestion and transfected into 293A cells by lipofectamine-2000 (Life Technologies) according to the protocol recommended by the manufacturer. On day 2 after transfection, the 293A cells were passaged and cultivated until 80% of cells became detached. The cell suspension was frozen then thawed three times. After centrifugation at 1,750 x *g* for 15 min, the supernatant was used as the gene expression adenoviral preparation (*Ad-EfnB1*). The titers for the adenoviral preparation were approximately 2×10^8^ plaque forming units (pfu)/mL. In these experiments, the adenovirus expressing β-galactosidase (*Ad-βgal*) was used as a control.

### Infection of adipocytes with the prepared adenovirus

For efficient transduction of the adenovirus, 3T3-L1 cells stably expressing Coxsackie-Adenovirus Receptor (CAR-3T3-L1) were used in the adenoviral study [18,19]. On day 7 after the induction of differentiation, CAR-3T3-L1 adipocytes were infected with *Ad-EfnB1* or *Ad-βgal* at 2.0 multiplicity of infection (MOI) (Figure S2). At 24 hrs after adenovirus infection, the medium was changed to remove uninfected adenovirus. At day 9, CAR-3T3-L1 adipocytes were cocultured with RAW264.7 cells using the transwell inserts, as described above. After co-incubation for 24 hrs, CAR-3T3-L1 adipocytes were harvested and subjected to quantitative RT-PCR or immunoblotting.

### Quantitative RT-PCR

Isolation of total RNA and production of cDNA were performed as described previously [17]. RT-PCR was performed on the ViiATM 7 real-time PCR system (Life Technologies) using the THUNDERBIRDTM qPCR Mix (TOYOBO, Osaka, Japan) according to the instructions provided by the manufacturer. For quantitative precision, the same amount of total RNA was consistently used for each expression analysis and the expression level of each gene was normalized by the mRNA level of a housekeeping gene, ribosomal protein, large, P0 (*Rplp0/36B4*). The following is a list of the primers used in this study: mouse *Ephrin-B1*, 5’-ATT ACA TCA ACG TCC AAT GGG AG-3’ and 5’-CCC AAC CTT CAT AAC GAT CTT CA-3’; mouse *Mcp-1*, 5’-CCA CTC ACC TGC TGC TAC TCA T-3’ and 5’-TGG TGA TCC TCT TGT AGC TCT CC-3’; mouse *Il-6*, 5’-GAG GAT ACC ACT CCC AAC AGA CC-3’; mouse *Adiponectin*, 5’- GAT GGC AGA GAT GGC ACT CC-3’; mouse *Mcp-3*, 5’- GCT GCT TTC AGC ATC CAA GTG-3’ and 5’- CCA GGG ACA CCG ACT ACT G-3’; mouse *Rplp0/36B4*, 5’- AAG CGC GTC CTG GCA TTG TCT-3’ and 5’- CCG CAG GGG CAG CAG TGG T-3’.

### Monocyte adherent assay

The adhesion of THP-1 human monocytic cell line to 3T3-L1 adipocytes was determined as described previously with minor modification [20]. Briefly, THP-1 cells were fluorescently labeled by incubation with calcein-AM (Dojin Chemical, Kumamoto, Japan) for 45 min, and washed twice in RPMI medium. 3T3-L1 adipocytes transfected with siRNA for *EfnB1* or negative control were incubated with TNF-α (1 ng/mL) for 24 hrs and CAR-3T3-L1 adipocytes infected with *Ad-EfnB1* or *Ad-βgal* were cocultured with RAW264.7 cells for 24 hrs prior to the adhesion assay. The labeled THP-1 cells were added to 3T3-L1 adipocytes and allowed to adhere for 90 min at 4°C. Cells were washed gently three times to remove non-adherent monocytes. Adherent cells were lysed with 50 mmol/L Tris (pH 8.4)/0.1% SDS, and fluorescence was measured with excitation at 485 nm wavelength and detection at 535 nm wavelength.

### Immunoblotting

Preparation of protein extracts from tissues and cells was performed as described previously [21]. For measurement of extracellular signal-regulated kinase (ERK) 1/2 phosphorylation, CAR-3T3-L1 adipocytes were infected with *Ad-EfnB1* or *Ad-βgal* before the assay. After 24-hr incubation, cells were incubated with or without TNF-α (1 ng/mL) for 5 min. At the end of incubation, the cells were harvested and subjected to immunoblotting. Twenty µg of protein was subjected to 4-20% gradient SDS-PAGE gel and then transferred to a nitrocellulose membrane (GE Healthcare, Little Chalfont, UK). For immunoblotting, the membrane was incubated with 1:1,000 dilution of goat anti-ephrin-B1 (R&D systems Inc., Minneapolis, MN), rabbit anti-α-tubulin, mouse anti-phospho-Erk1/2, or rabbit ant- Erk1/2 (Cell signaling technology, Danvers, MA). Detection was achieved using the enhanced chemiluminescence kit (GE Healthcare).

### Statistical analysis

All values were expressed as mean±SD. Differences between groups were analyzed by one-factor ANOVA and unpaired Student’s *t*-test. *P* values less than 0.05 were considered statistically significant.

## Results

### Changes in Ephrin-B1 expression in obese adipose tissue

**Figure 1:**
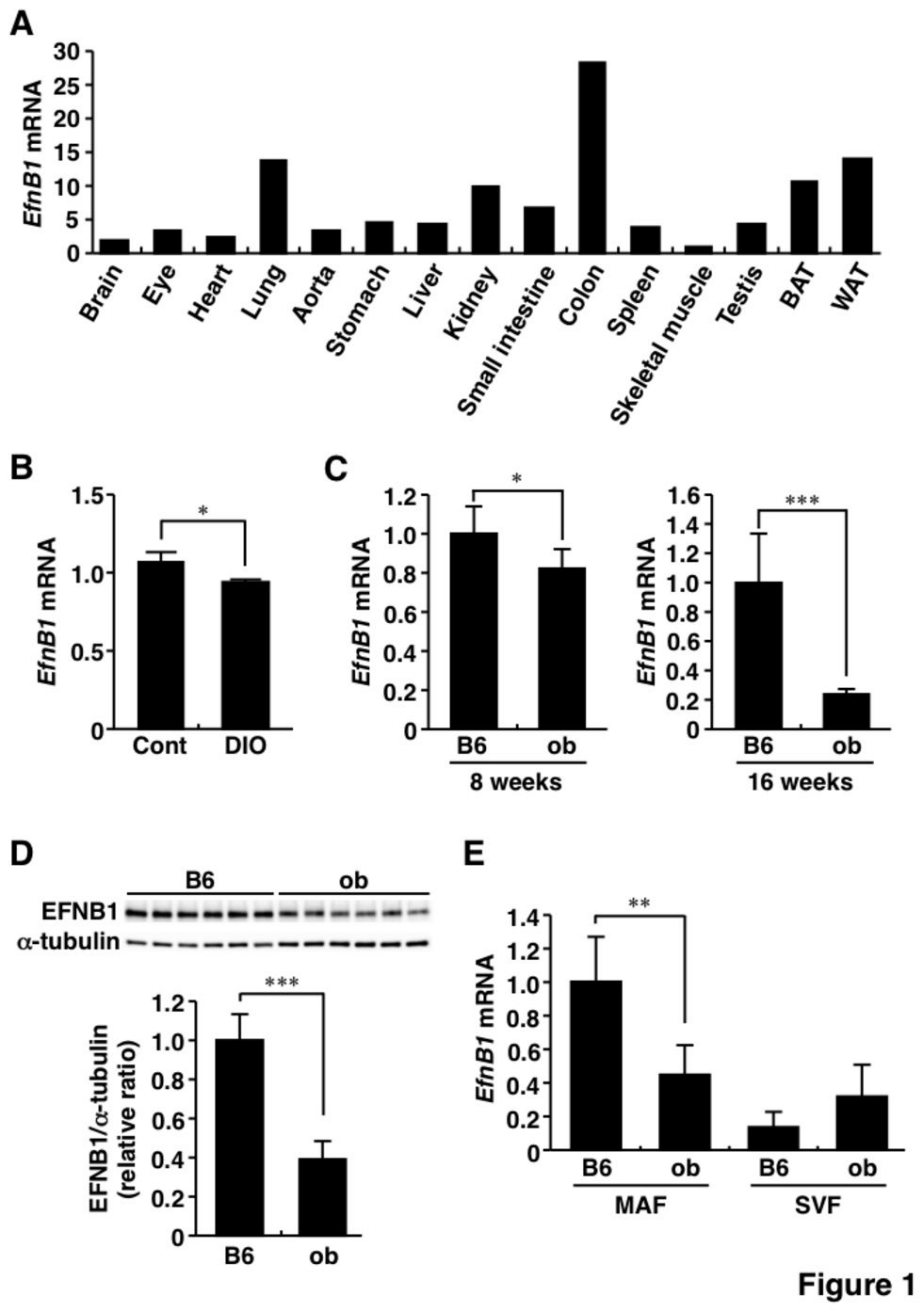
Expression of Ephrin-B1 in obese adipose tissue. A, Tissue distribution of Ephrin-B1 mRNA. Experiments were conducted in C57BL/6N mice under 12 hrs-fasting state at 12 weeks of age. B, Changes in adipose Ephrin-B1 mRNA level under high-fat/high-sucrose (HF/HS) diet. C57BL/6N mice were fed normal chaw diet (Cont) or HF/HS from 8 to 16 weeks of age. n=3 for each group. C, Adipose Ephrin-B1 mRNA levels in obese model mice. Ephrin-B1 mRNA level in WAT was examined in C57BL/6N (B6) and ob/ob (ob) mice at 8 and 16 weeks of age, respectively. n=6 for each group. D, Changes in adipose Ephrin-B1 protein level in mice of the obese model. Immunoblotting was performed using WAT of 16-week-old B6 and ob mice. Relative protein level (Ephrin-B1/α-tubulin) was calculated by densitometry. n=6 for each group. E, Ephrin-B1 mRNA level in fractionated WAT. WAT of 16-week-old B6 and ob mice was separated into mature adipocytes fraction (MAF) and stromal vascular fraction (SVF) as described in Materials and Methods section. n=5-6 for each group. *EfnB1* and EFNB1, Ephrin-B1; WAT, white adipose tissue; BAT, brown adipose tissue; DIO, diet-induced obesity; B6, C57BL/6J mice; ob, *ob/ob* mice; MAF, mature adipocytes fraction; SVF, stromal vascular fraction. Values are mean±SD. *P<0.05; **P<0.01; ***P<0.001.

*EFNB1* mRNA levels in peripheral blood cells correlated negatively with the estimated visceral fat area (eVFA) in human subjects (range of BMI, 25.4-51.2 kg/m^2^; range of eVFA, 80-386 cm^2^) (Figure S3). The significance of EFNB1 in adipose tissue was analyzed in mice and cell experiments. Figure 1A shows the tissue distribution of *EfnB1* mRNA in lean control (C57BL/6) mice. *EfnB1* mRNA was highly expressed in the colon and lung, and also abundantly detected in WAT.

Next, we examined changes in *EfnB1* mRNA level in obesity. The *EfnB1* mRNA level in WAT was significantly lower in mice fed HF/HS diet than the normal chow group (Figure 1B). Furthermore, adipose tissue *EfnB1* mRNA level was significantly lower in obese *ob/ob* mice at 8 and 16 weeks of age, compared with that in lean control mice at the corresponding ages (Figure 1C). The amount of EFNB1 protein was also lower in *ob/ob* mice compared to lean control mice at 16 weeks of age (Figure 1D). Next, we examined *EfnB1* mRNA level in MAF and SVF following fractionation of adipose tissue (Figure 1E). *EfnB1* mRNA was abundantly expressed in MAF compared to SVF and was significantly lower in obese MAF. These results indicate that under-expression of *EfnB1* mRNA in adipocytes may account for the low *EfnB1* mRNA level in obese WAT.

We also investigated the effects of nutritional changes on adipose tissue *EfnB1* mRNA level. Fasting increased *EfnB1* mRNA level in WAT (Figure S4a), but streptozotocin (STZ)-induced insulin-deficiency had no influences on the mRNA level (Figure S4b), suggesting no direct effect for insulin on *EfnB1* mRNA level in WAT.

### Changes in Ephrin-B1 expression in 3T3-L1 adipocytes

**Figure 2:**
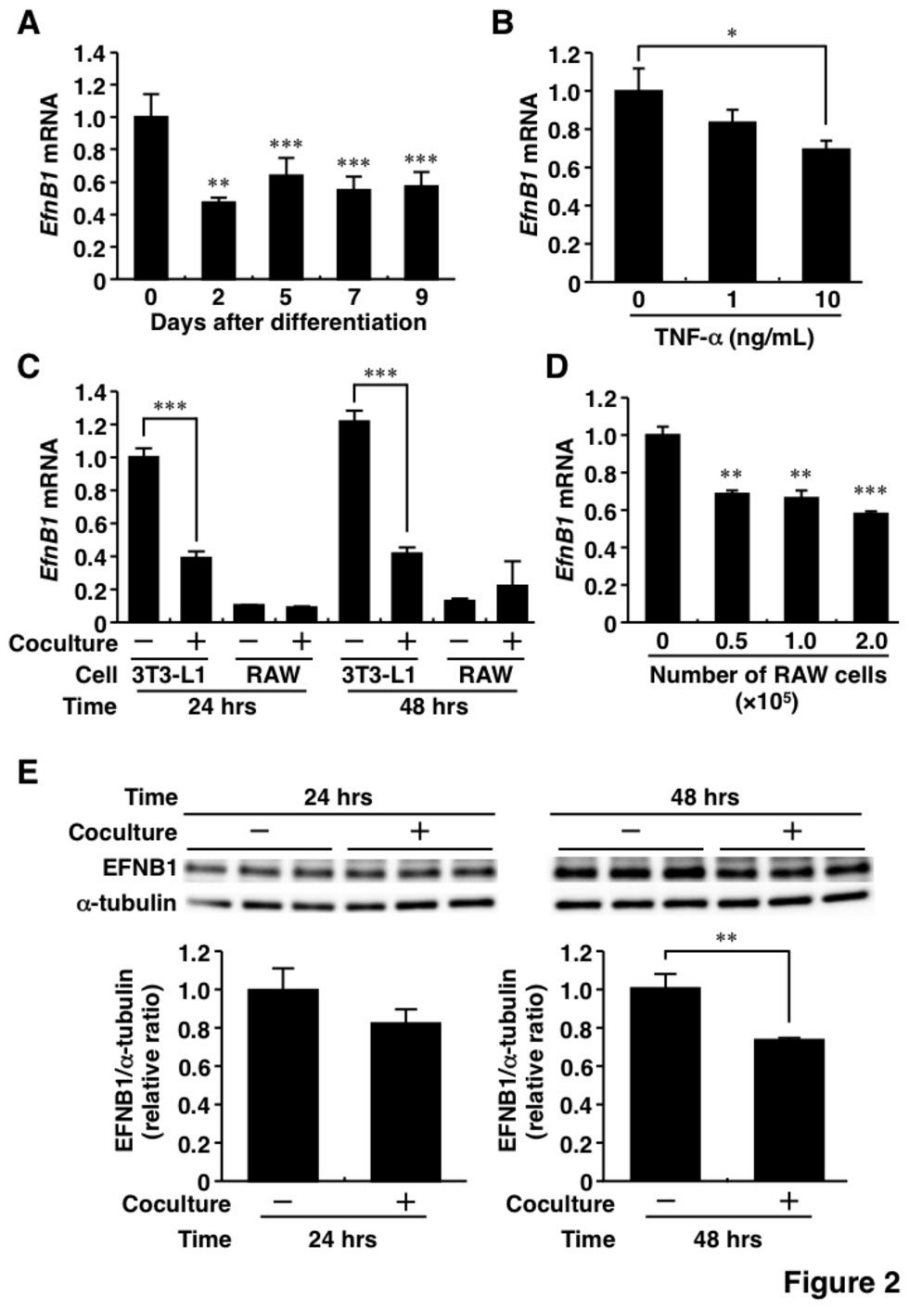
Regulation of Ephrin-B1 in 3T3-L1 adipocytes. A, Changes in Ephrin-B1 mRNA level during differentiation of 3T3-L1 adipocytes. B, Effect of tumor necrosis factor-α (TNF-α) on Ephrin-B1 mRNA level in 3T3-L1 adipocytes. 3T3-L1 adipocytes were co-incubated for 24 hrs in the absence or presence of TNF-α at the indicated concentrations. C, Changes in Ephrin-B1 mRNA level under the coculture of 3T3-L1 adipocytes and RAW264.7 cells. The co-culture system was based on the transwell method as described in the Materials and Methods section. Ephrin-B1 mRNA levels were measured in 3T3-L1 adipocytes and RAW264.7 cells cultured alone or together. D, Effects of co-culture of 3T3-L1 adipocytes with RAW264.7 cells on Ephrin-B1 mRNA level in 3T3-L1 adipocytes. 3T3-L1 adipocytes were cocultured for 24 hrs with the indicated number of RAW264.7 cells. E, Ephrin-B1 protein level in 3T3-L1 adipocytes cocultured with RAW264.7 cells. 3T3-L1 adipocytes were cocultured with RAW264.7 cells through transwell and collected at 24 and 48 hrs after coculture. Western blotting was performed using the indicated antibodies. The relative protein level (Ephrin-B1/α-tubulin) was calculated by densitometry. *EfnB1* and EFNB1, Ephrin-B1; TNF-α, tumor necrosis factor-α; RAW, RAW264.7 cells. Values are mean±SD; n=3 for each group. *P<0.05; **P<0.01; ***P<0.001.

Since *EfnB1* was mainly expressed in mature adipocytes (Figure 1E), we examined the regulation of *EfnB1* using 3T3-L1 adipocytes. *EfnB1* mRNA level decreased during differentiation of 3T3-L1 cells into adipocytes (Figure 2A). To investigate the regulation of *EfnB1* in WAT of obese animals, 3T3-L1 adipocytes were stimulated with tumor necrosis factor-α (TNF-α) and cocultured with RAW264.7 macrophages (Figure S1). As shown in Figure 2B, TNF-α reduced *EfnB1* mRNA level in 3T3-L1 adipocytes and the effect was dose-dependent. Interestingly, *EfnB1* mRNA level was markedly reduced in 3T3-L1 adipocytes after coculture of these cells with RAW264.7 cells, while the level in RAW264.7 cells was low and not influenced by co-culture with 3T3-L1 adipocytes (Figure 2C). *EfnB1* mRNA level significantly decreased in 3T3-L1 adipocytes in proportion with the number of RAW264.7 cells (Figure 2D). Furthermore, the amount of EFNB1 protein also decreased in 3T3-L1 adipocytes under co-culture with RAW264.7 cells (Figure 2E). Collectively, the results of these *in vitro* experiments suggest that macrophage-derived factors, e.g., TNF-α, suppress adipose EFNB1 expression.

### Effects of suppression and overexpression of Ephrin-B1 on adipose inflammatory response

**Figure 3:**
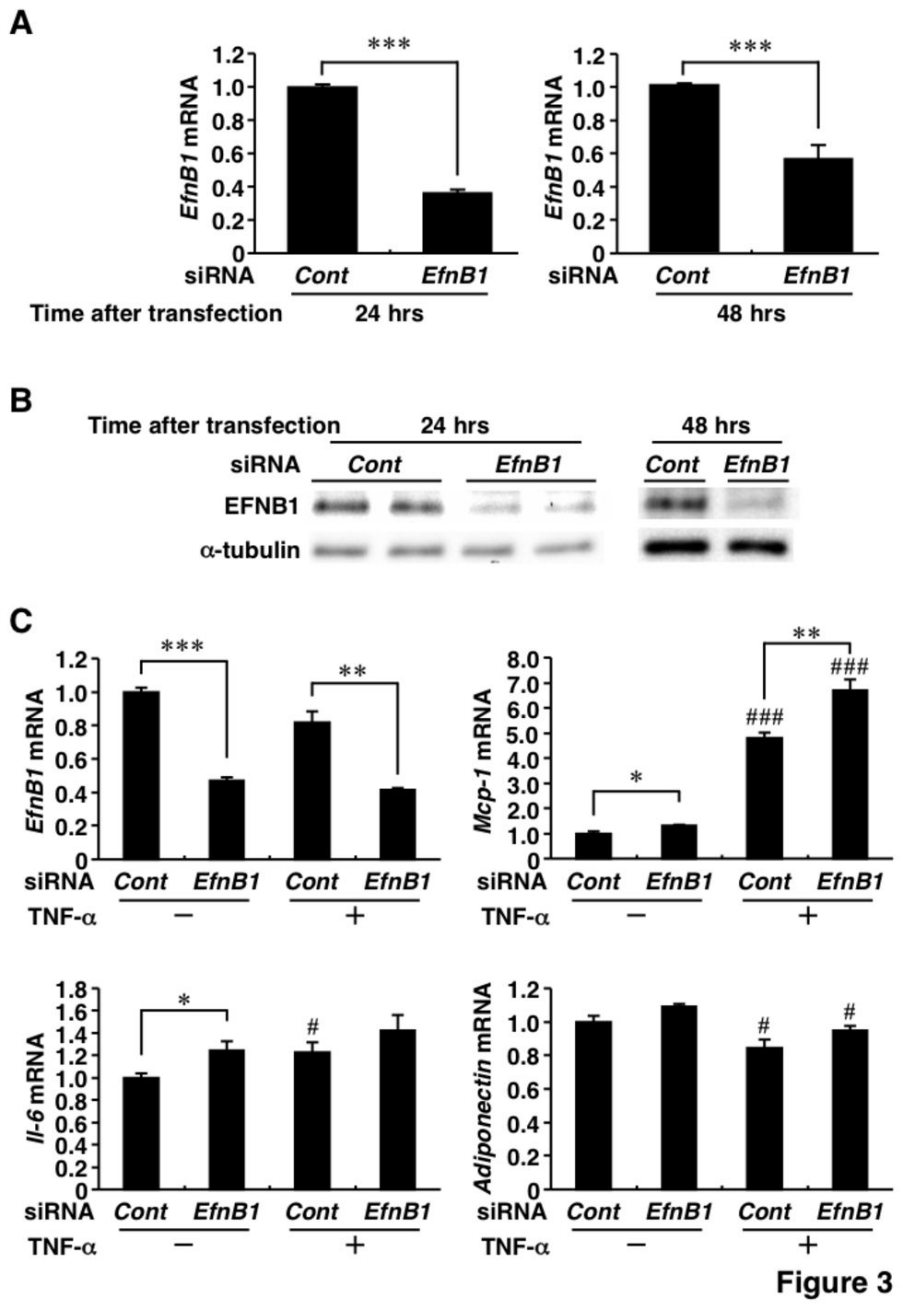
Inflammatory response in Ephrin-B1-knockdown 3T3-L1 adipocytes. The small inhibitory RNA (siRNA) for Ephrin-B1 was introduced in 3T3-L1 adipocytes as described in the Materials and Methods section. A and B, Ephrin-B1 mRNA (A) and protein (B) levels at 24 hrs (left) and 48 hrs (right) after transfection of control-siRNA or Ephrin-B1-siRNA. C, Changes in adipocytokine mRNA levels by Ephrin-B1-siRNA. 3T3-L1 adipocytes were incubated with or without 1 ng/mL of tumor necrosis factor-α (TNF-α) for 24 hrs after siRNA transfection. *EfnB1* and EFNB1, Ephrin-B1; TNF-α, tumor necrosis factor-α; *Mcp-1*, monocyte chemoattractant protein-1; *Il-6*, interleukin-6. Values are mean±SD; n=3 for each group. *P<0.05; **P<0.01; ***P<0.001. #*P*<0.05; # # #*P*<0.001, compared to TNF-α (-).

To explore the role of EFNB1 in adipocytes, knockdown and overexpression of EFNB1 were conducted by using siRNA and adenovirus, respectively. The introduction of siRNA designed for *EfnB1* (*EfnB1-siRNA*) successfully reduced *EfnB1* mRNA (Figure 3A) and EFNB1 protein (Figure 3B) levels. Interestingly, *Mcp-1* mRNA level was significantly increased with and without TNF-α, when 3T3-L1 adipocytes were transfected with *EfnB1-siRNA* (Figure 3C). The introduction of *EfnB1-siRNA* caused a significant increase in *Il-6* mRNA level in TNF-α-unstimulated 3T3-L1 adipocytes (Figure 3C). However, *EfnB1-siRNA* had no effect on *Adiponectin* mRNA (Figure 3C).

**Figure 4:**
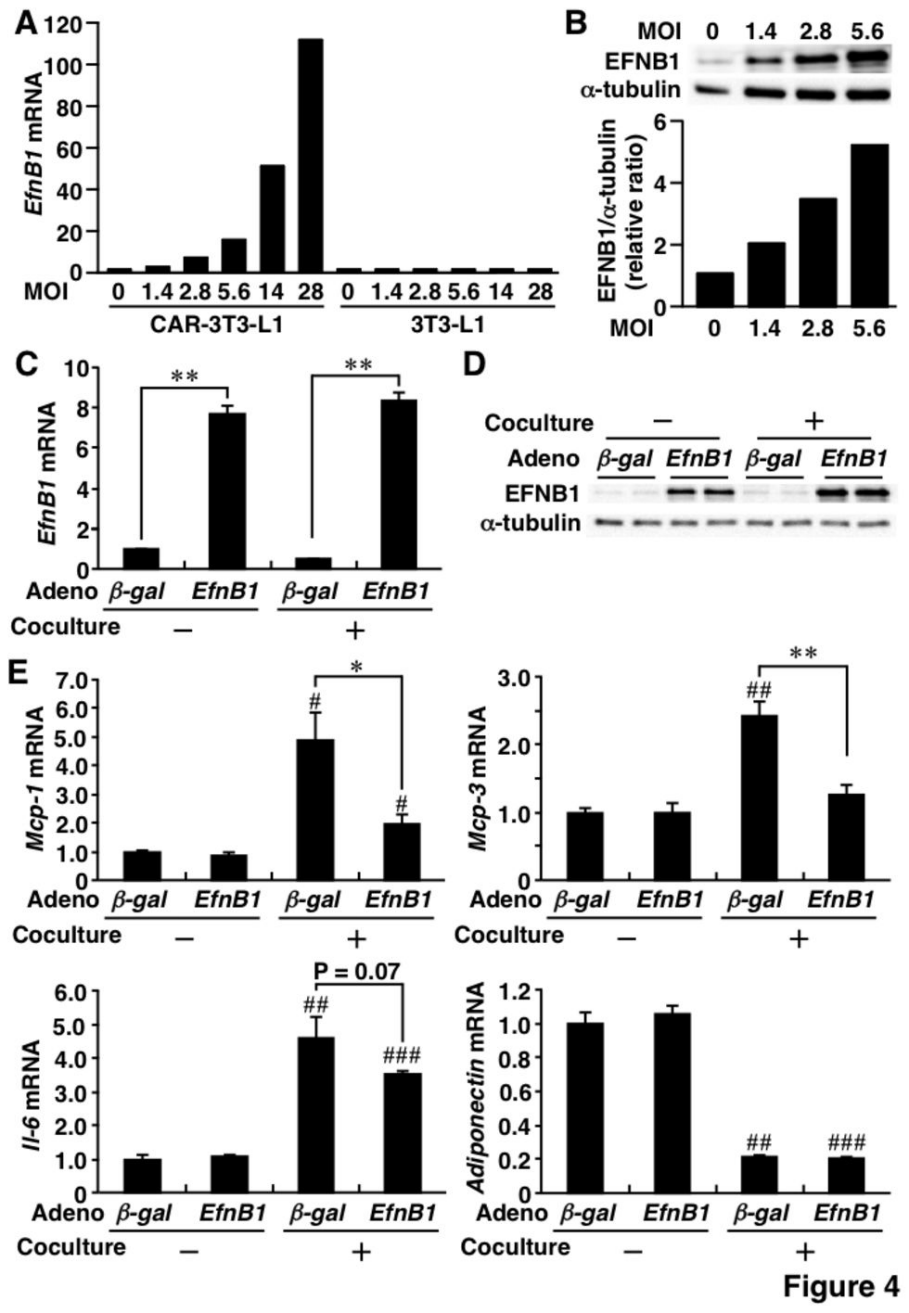
Suppression of inflammatory response by adenovirus-mediated overexpression of Ephrin-B1 in 3T3-L1 adipocytes. CAR-3T3-L1 preadipocytes and adipocytes were infected with adenovirus expressing Ephrin-B1 (Ad-EfnB1) or β-galactosidase (Ad-βgal). CAR-3T3-L1 adipocytes were transfected with *Ad-EfnB1* or *Ad-βgal* on day 7 after differentiation and cocultured with RAW264.7 cells using transwell inserts on day 9. After coincubation with RAW264.7 cells for 24 hrs, CAR-3T3-L1 adipocytes were collected and analyzed. A, Expression levels of Ephrin-B1 mRNA in CAR-3T3-L1 preadipocytes (left) and 3T3-L1 preadipocytes (right) following *Ad-EfnB1* transfection. B, Dose-response of Ephrin-B1 protein level by *Ad-EfnB1* infection in CAR-3T3-L1 preadipocytes. The relative protein level (Ephrin-B1/α-tubulin) was calculated by densitometry. C and D, Adenoviral-mediated overexpression of Ephrin-B1 mRNA (C) and protein (D) levels in CAR-3T3-L1 adipocytes cultured with or without RAW264.7 cells. E, Changes in adipocytokine mRNA levels by overexpression of Ephrin-B1 in CAR-3T3-L1 adipocytes. *EfnB1* and EFNB1, Ephrin-B1; CAR-3T3-L1, 3T3-L1 cells stably expressing Coxsackie-Adenovirus Receptor; MOI, multiplicity of infection; *Mcp-1*, monocyte chemoattractant protein-1; *Il-6*, interleukin-6; *Mcp-3*, monocyte chemoattractant protein-3. Values are mean±SD; n=3 for each group. *P<0.05; **P<0.01. #*P*<0.05; # #*P*<0.01; # # #*P*<0.001, compared to coculture (-).

Experiments designed to examine the effects of overexpression of EFNB1 were performed using adenovirus expressing *EfnB1* (*Ad-EfnB1*) as described in Materials and Methods section. Transfection with *Ad-EfnB1* successfully and dose-dependently increased *EfnB1* mRNA (Figure 4A) and EFNB1 protein (Figure 4B) levels in CAR-3T3-L1 preadipocytes, but did not in 3T3-L1 preadipocytes (Figure 4A). Following transfection of CAR-3T3-L1 adipocytes with *Ad-EfnB1*, the cells were cocultured with RAW264.7 cells (Figure S2). *EfnB1* mRNA (Figure 4C) and EFNB1 protein (Figure 4D) was substantially overexpressed in CAR-3T3-L1 adipocytes with or without co-culture with RAW264.7 cells. Figure 4E shows changes in *EfnB1* mRNA related to adipose inflammation. High mRNA levels of *Mcp-1* and *Mcp-3* were noted in CAR-3T3-L1 adipocytes following coculture with RAW264.7 cells, while such increase was markedly suppressed following transfection with *Ad-EfnB1*. The coculture-related increase in *Il-6* mRNA level tended to diminish in CAR-3T3-L1 adipocytes transfected with *Ad-EfnB1*. Similar to the siRNA study, transfection with *Ad-EfnB1* did not alter *Adiponectin* mRNA level in CAR-3T3-L1 adipocytes.

### Effects of Ephrin-B1 on monocyte adhesion and activation of extracellular signal-regulated kinase 1/2

**Figure 5:**
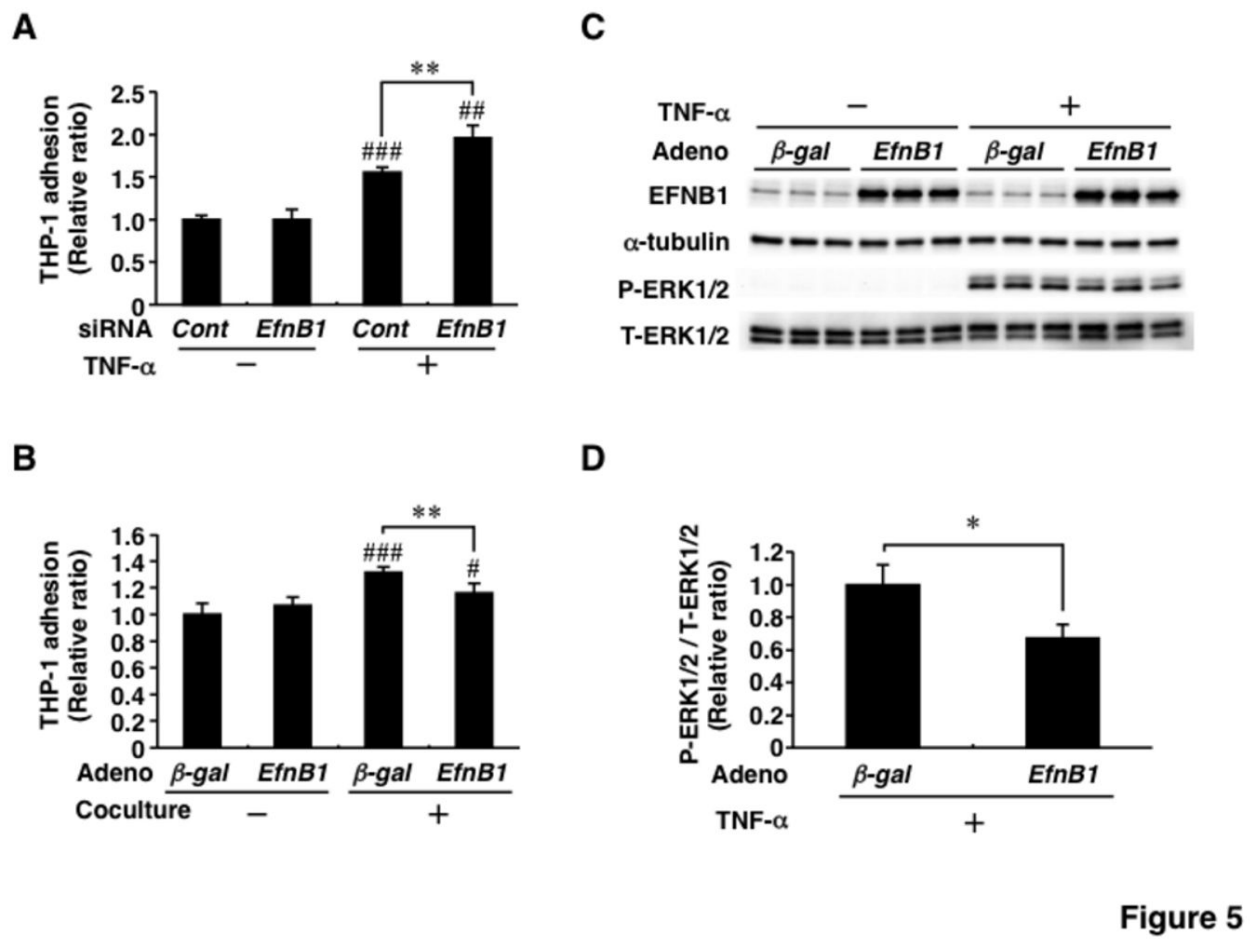
Effects of Ephrin-B1 on monocyte adhesion to adipocytes and activation of extracellular signal-regulated kinase. For the monocyte adhesion assay, fluorescently labeled THP-1 cells were added to adipocytes and coincubated for 90 min. After removal of nonadherent monocytes, the intensity of fluorescence was measured as described in the Materials and Methods section. A, Effect of Ephrin-B1-knockdown on monocyte adhesion to 3T3-L1 adipocytes. 3T3-L1 adipocytes were transfected with siRNA for Ephrin-B1 (EfnB1) or negative control (Cont) and then the cells were incubated in the absence or presence of tumor necrosis factor-α (TNF-α) at 1 ng/mL for 24 hrs prior to adherent assay. n=6 for each group. B, Effect of overexpression of Ephrin-B1 on monocyte adhesion to CAR-3T3-L1 adipocytes. CAR-3T3-L1 adipocytes were transfected with an adenovirus expressing Ephrin-B1 (Ad-EfnB1) or β-galactosidase (Ad-βgal). The transfected adipocytes were cocultured with RAW264.7 cells for 24 hrs before the adhesion assay. n=6 for each group. C, Representative blots of ERK signal in the adenovirus study. Western blotting was performed using antibodies against Ephrin-B1 (EFNB1), α-tubulin, phosphorylated extracellular signal-regulated kinase 1/2 (P-ERK1/2), and total extracellular signal-regulated kinase 1/2 (T-ERK1/2). After 24 hrs of transfection with *Ad-EfnB1* or *Ad-βgal*, CAR-3T3-L1 adipocytes were stimulated with or without 1 ng/mL of TNF-α for 5 min and subjected to immunoblotting. n=3 for each group. D, Phosphorylation of ERK1/2. The relative signal intensity for P-ERK1/2 and T-ERK1/2 was calculated by densitometry and expressed as phosphorylation of ERK. n=3 for each group. *EfnB1* and EFNB1, Ephrin-B1; CAR-3T3-L1, 3T3-L1 cells stably expressing Coxsackie-Adenovirus Receptor; TNF-α, tumor necrosis factor-α. Values are mean±SD. **P<0.01. #*P*<0.05; # #*P*<0.01; # # #*P*<0.001, compared to TNF-α (-) or coculture (-).

*Mcp-1* and *Mcp-3* mRNA levels clearly changed following suppression and overexpression of EFNB1 (Figures 3C and 4E). We hypothesized that adipose EFNB1 contributes to monocyte recruitment into adipose tissues. The hypothesis was tested using monocyte adherent assay and THP-1 cells as described in Materials and Methods section (Figure 5A and B). Stimulation of 3T3-L1 adipocytes with TNF-α increased the adhesion of THP-1 cells and such increase was significantly augmented by the introduction of *EfnB1-siRNA* (Figure 5A). Adhesion of THP-1 cells to *Ad-βgal*-infected-CAR-3T3-L1 adipocytes was slightly but significantly increased following coculture with RAW264.7 cells (Figure 5B). Such coculture-related increase of monocyte adhesion was significantly blunted by transfection of *Ad-EfnB1* (Figure 5B).

Finally, we examined the effect of EFNB1 on the activation of ERK1/2, which is one of the major signaling pathways in adipose inflammatory process [7-10]. In *Ad-βgal*-infected-CAR-3T3-L1 adipocytes, stimulation with TNF-α caused phosphorylation of ERK1/2 (Figure 5C). Furthermore, transfection of the adenovirus significantly reduced TNF-α-induced phosphorylation of ERK1/2 (Figure 5C and D).

## Discussion

The main findings of the present study were: *(1*) expression of EFNB1 in WAT and the expression level was significantly low in obese mature adipocytes(2). EFNB1 expression in 3T3-L1 adipocytes was significantly reduced by TNF-α, and markedly suppressed by coculture of 3T3-L1 adipocytes with RAW264.7 cells(3). Adipose EFNB1 exhibited anti-inflammatory properties and inhibited monocyte adhesion to adipocytes(4). EFNB1 in adipocytes suppressed TNF-α-mediated ERK1/2 activation.

The present study demonstrated for the first time a significantly low level of EFNB1 expression in obese fat tissue. Regulation of EFNB1 has remained uncertain and has not been examined in adipocytes. TNF-α, which is increased in obesity, significantly reduced *EfnB1* mRNA expression level (Figure 2B), suggesting that this cytokine is one of factors accounting for the low expression of EFNB1 in obese adipose tissue. Interestingly, coculture with RAW264.7 macrophages cells definitely suppressed EFNB1 level in adipocytes (Figure 2C-E). The coculture-related reduction of EFNB1 was larger than TNF-α-mediated decrease in *EfnB1*, indicating that macrophage-derived undetermined factors can modulate EFNB1 expression in adipocytes.

The Eph-ephrin system is important in neural tissue development, plasticity, and regeneration, immune function, bone homeostasis, and various types of cancers [15]. However, the role of Eph-ephrin system in various metabolic diseases (e.g., diabetes, atherosclerosis, and obesity) remains poorly understood. Konstantinova et al [22] demonstrated that β-cells communicated through EphA-ephrin-A and its signaling controls insulin secretion in response to glucose levels. In other studies, EFNB1 was detected in T-lymphocytes and macrophages in atherosclerotic lesions of the human aorta [23,24]. Interestingly, EFNB1 inhibited MCP-1-dependent monocyte migration, although the role of this phenomenon in atherosclerogenesis remains to be defined. In the present study, EFNB1-overexpression clearly suppressed the elevation of *Mcp-1*, *Mcp-3*, and *Il-6* mRNA levels in adipocytes when they were cocultured with RAW 264.7 macrophages, while knockdown of EFNB1 increased *Mcp-1* in adipocytes. These results suggest that EFNB1 under-expression in obese WAT accelerates the infiltration of monocytes/macrophages into WAT.

Chronic low-grade inflammation is observed in obese adipose tissue and is closely associated with the metabolic syndrome. Several metabolic stresses, such as stress of the endoplasmic reticulum [25], oxidative stress [26], and hypoxia [27] may induce adipose inflammation and adipocyte dysfunction, which can cause disorders of circulating fatty acids, accumulation of reactive oxygen species, and hyperadipocytokinemia, located upstream in the development of the metabolic syndrome and atherosclerosis [2,28,29]. Importantly, various adipocytes-derived chemokines increase monocyte recruitment into adipose tissue and the MCP-1/CCR2 pathway is known to play a crucial role in monocyte/macrophage infiltration into obese adipose tissue [30-32], suggesting the involvement of adipose MCP-1 in the prevention of the metabolic syndrome. Inflammatory cytokines, such as TNF-α, interleukin-1β (IL-1β), and transforming growth factor-β (TGF-β), increase MCP-1 through the activation of ERK, nuclear factor-κB (NFκB), and c-Jun N-terminal kinase (JNK) pathway [33-35]. In the course of adipocyte hypertrophy, ERK is activated while mitogen-activated protein kinase phosphatase-1 (MKP-1) is inactivated [18]. Inhibition of MKP-1 causes ERK activation and results in the elevation of MCP-1, indicating the significant role of MKP-1 in obese adipocytes [18]. Importantly, the EFNB1 signal suppresses ERK activation in several cells [36,37]. Taken together, our results suggest that adipose EFNB1 regulates MCP-1 expression through the ERK pathway, although further investigation is needed to confirm these findings.

**Figure 6:**
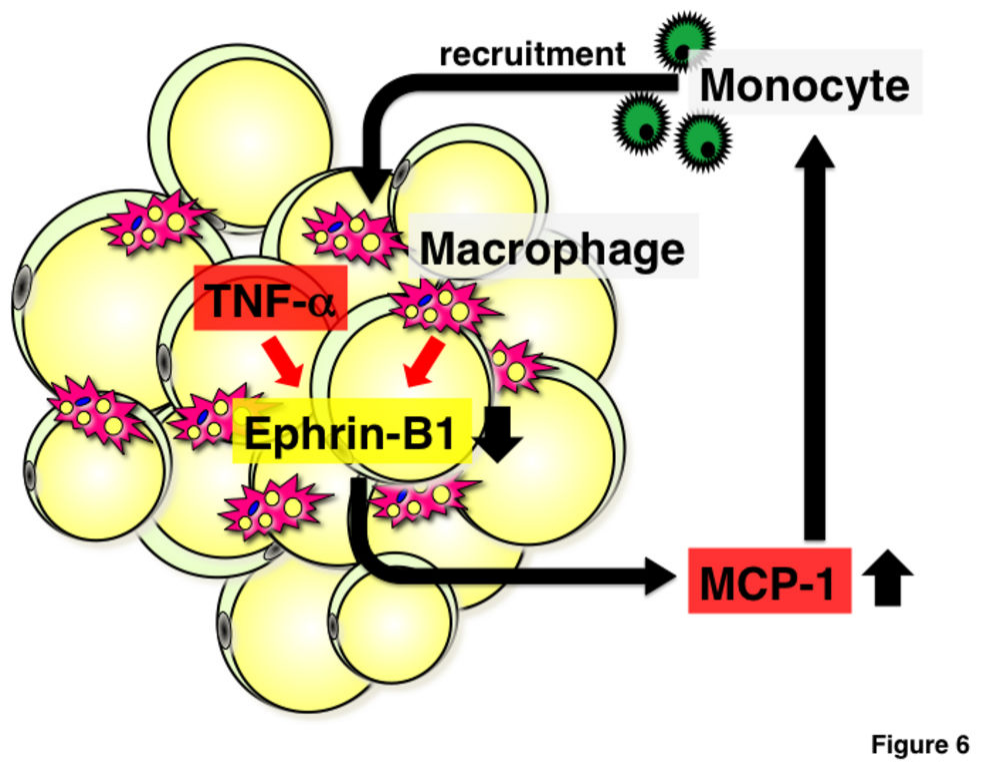
A schematic diagram illustrating the possible role of Ephrin-B1 in the development of adipose inflammation in obesity. In obese adipose tissues, TNF-α and macrophages repress Ephrin-B1 expression in adipocytes. Suppression of Ephrin-B1 in adipocytes augments MCP-1 expression and accelerates monocytes recruitment into adipose tissues. Ephrin-B1 may play an important role in adipose vicious cycle in obesity; reduction of adipose Ephrin-B1 expression in obesity could accelerate the vicious cycle involved in adipose tissue inflammation.

Figure 6 provides a model that summarizes the results of the present study. In obese adipose tissue, TNF-α and macrophage-derived undetermined factors suppress EFNB1 expression in adipocytes. Such under-expression results in augmentation of MCP-1 expression, which in turn increases the recruitment of monocytes into adipose tissues. Infiltrated monocytes may phenotypically change into macrophages and such macrophages reduce EFNB1 expression. Collectively, adipose EFNB1 serves as a suppressor of adipose inflammatory response. In obesity, under-expression of EFNB1 in adipose tissue may accelerate the vicious cycle of adipose tissue inflammation.

## Supporting Information

Figure S1 **Schematic illustration of cocultures of 3T3-L1 adipocytes and RAW264.7 macrophages.**
(PDF)

Figure S2 **Schematic illustration of adenovirus experiments of coculture of CAR-3T3-L1 adipocytes and RAW264.7 macrophages.** CAR-3T3-L1, 3T3-L1 cells stably expressing Coxsackie-Adenovirus Receptor; *Ad-EfnB1*, adenovirus expressing Ephrin-B1; *Ad-βgal*, adenovirus expressing β-galactosidase; m.c., medium change.
(PDF)

Figure S3 **Correlation between estimated visceral fat area (eVFA) and Ephrin-B1 mRNA level in peripheral blood cells.**
(PDF)

Figure S4 **Effects of nutritional changes on Ephrin-B1 mRNA level in WAT.** a. Effects of fasting and refeeding on Ephrin-B1 mRNA level. n=4 for each group. b. Effect of insulin-deficiency on Ephrin-B1 mRNA level. Control group; n=6, STZ group; n=11. *EfnB1*, Ephrin-B1; B6, C57BL/6N mice; ob, *ob/ob* mice; Cont, saline-treated mice; STZ, streptozotosin-treated mice. Values are mean±SD.
(PDF)
