# Supplementary figures and images for: A Novel Role for Adipose Ephrin-B1 in Inflammatory Response
Source: PLoS One. 2013 Oct 1;8(10):e76199. doi: 10.1371/journal.pone.0076199 (PMC3787942; doi:10.1371/journal.pone.0076199)

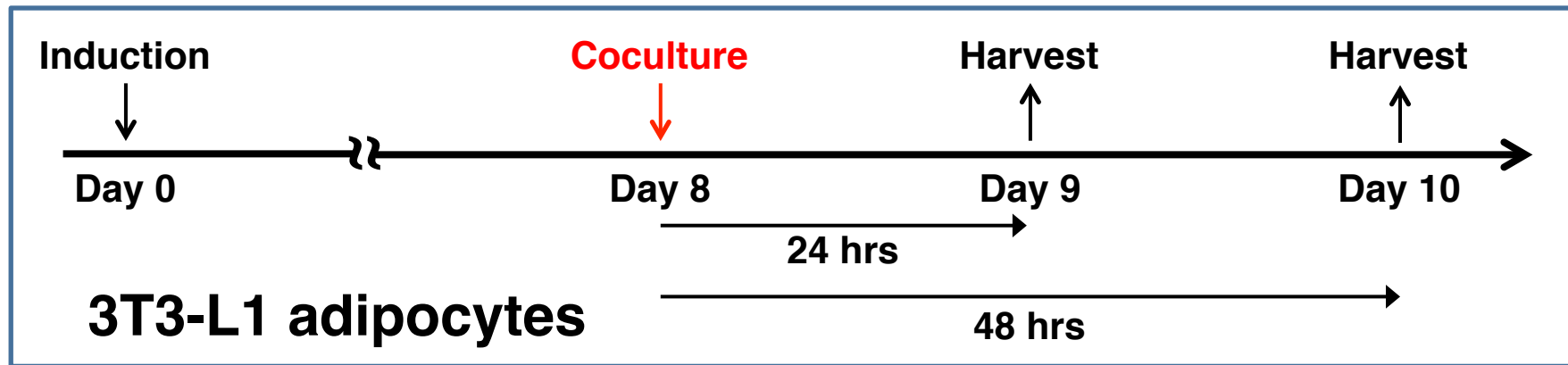

**3T3-L1 adipocytes**

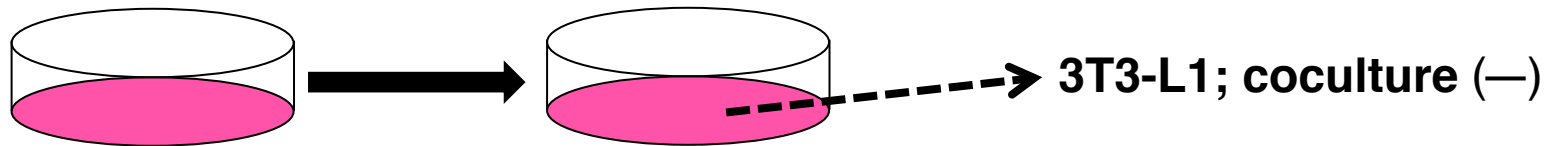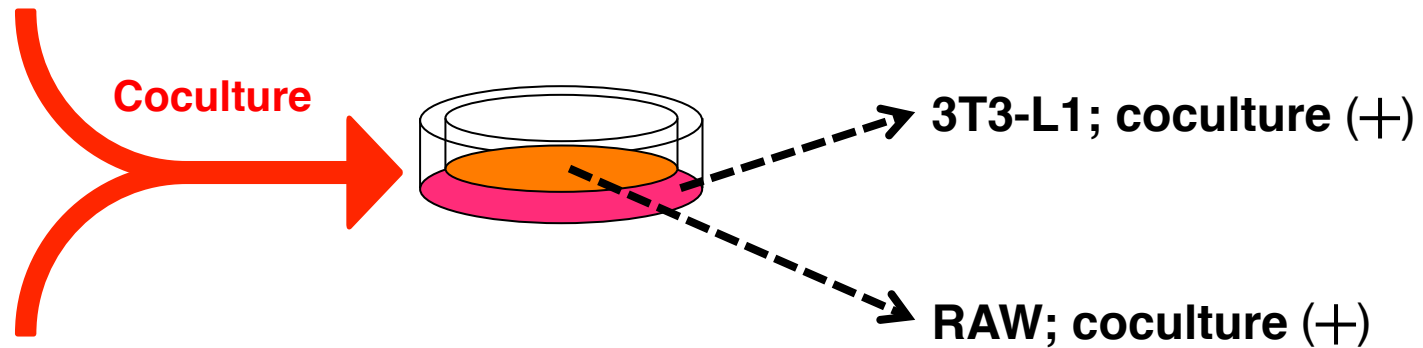

**RAW264.7 cells**

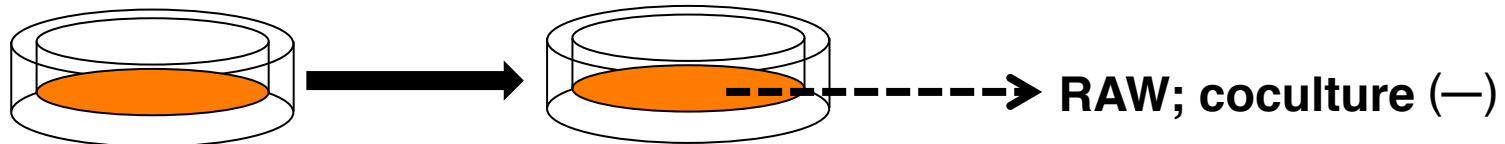

**Transwell plate**

**Figure S1**

Supplement: Figure S1 — Schematic illustration of cocultures of 3T3-L1 adipocytes and RAW264.7 macrophages. (PDF) [file pone.0076199.s001.pdf]

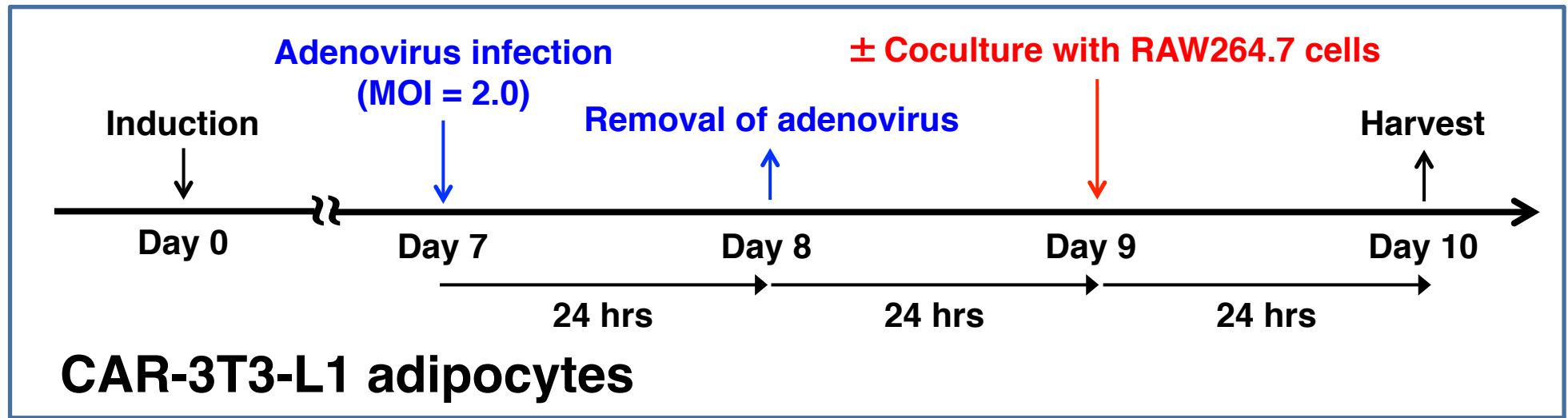

**CAR-3T3-L1 adipocytes**

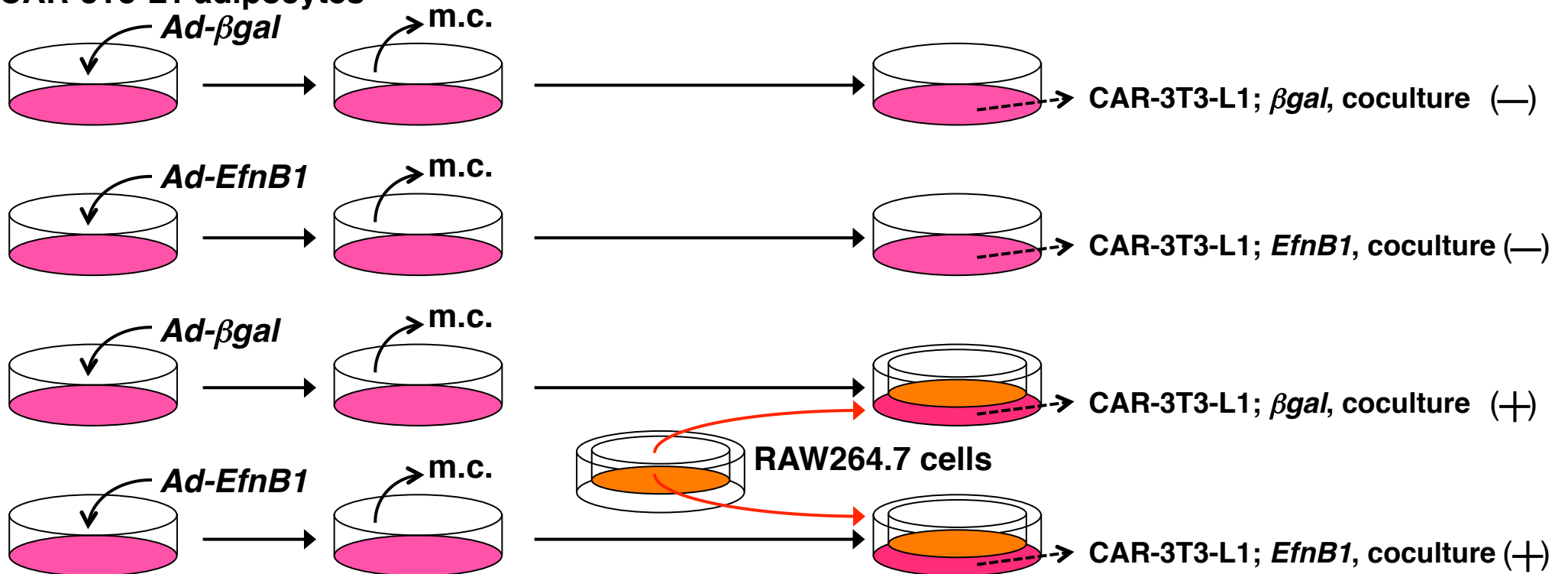

**Figure S2**

Supplement: Figure S2 — Schematic illustration of adenovirus experiments of coculture of CAR-3T3-L1 adipocytes and RAW264.7 macrophages. CAR-3T3-L1, 3T3-L1 cells stably expressing Coxsackie-Adenovirus Receptor; Ad-EfnB1, adenovirus expressing Ephrin-B1; Ad-βgal, adenovirus expressing β-galactosidase; m.c., medium change. (PDF) [file pone.0076199.s002.pdf]

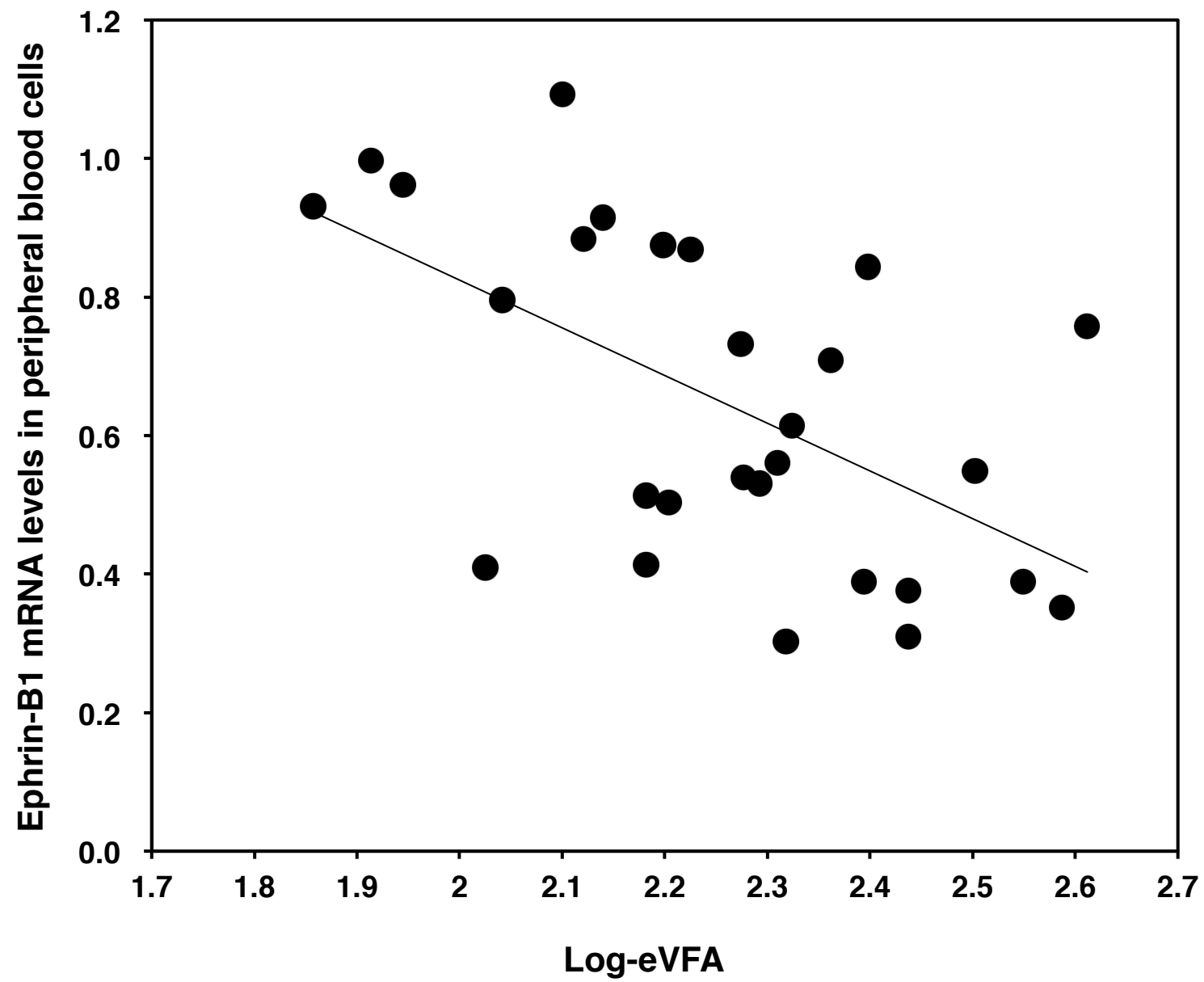

**Figure S3**

Supplement: Figure S3 — Correlation between estimated visceral fat area (eVFA) and Ephrin-B1 mRNA level in peripheral blood cells. (PDF) [file pone.0076199.s003.pdf]

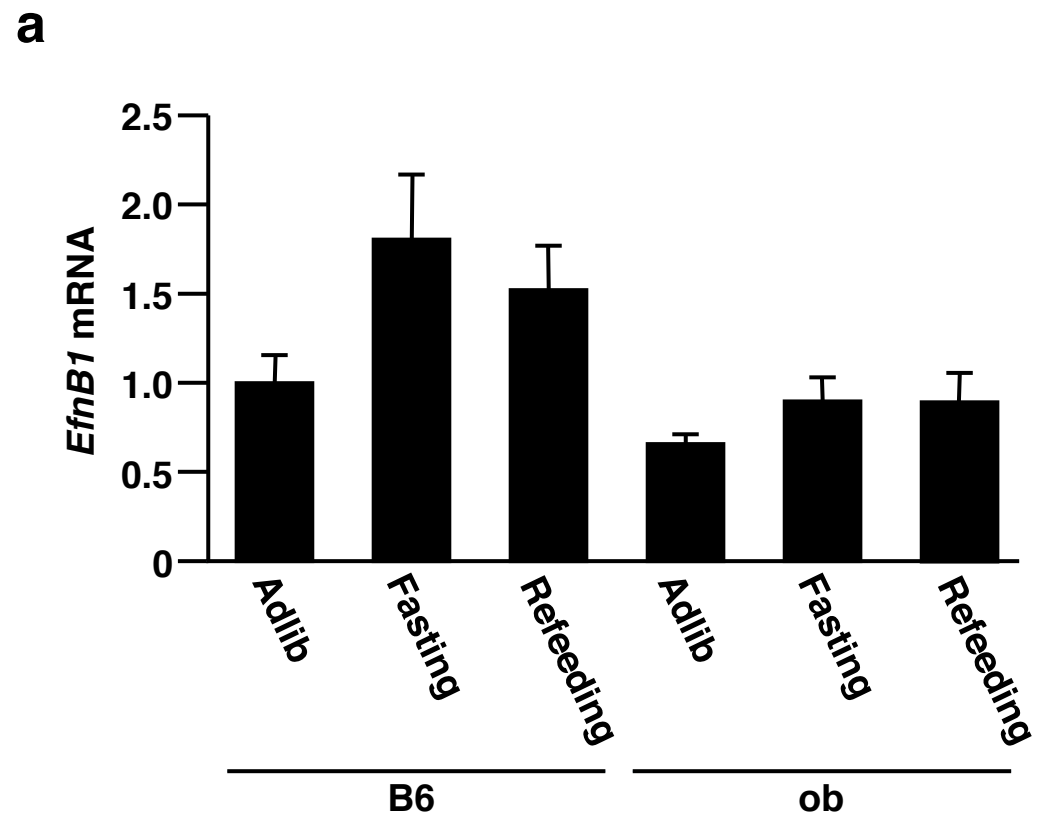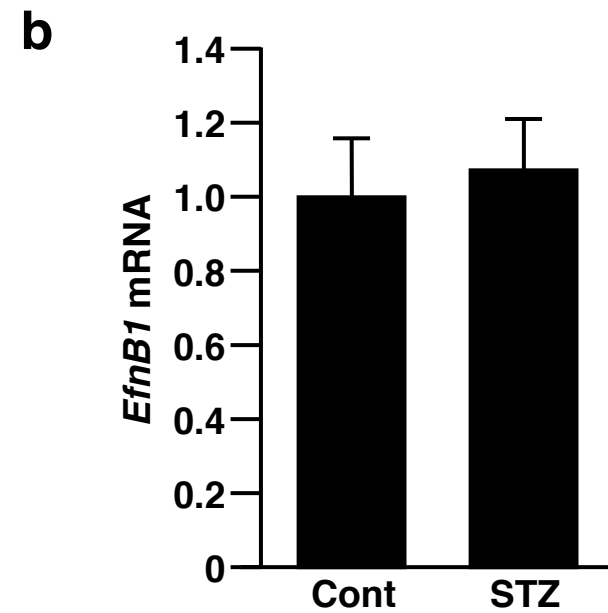

Figure S4

Supplement: Figure S4 — Effects of nutritional changes on Ephrin-B1 mRNA level in WAT. a. Effects of fasting and refeeding on Ephrin-B1 mRNA level. n=4 for each group. b. Effect of insulin-deficiency on Ephrin-B1 mRNA level. Control group; n=6, STZ group; n=11. EfnB1, Ephrin-B1; B6, C57BL/6N mice; ob, ob/ob mice; Cont, saline-treated mice; STZ, streptozotosin-treated mice. Values are mean±SD. (PDF) [file pone.0076199.s004.pdf]
